# Assessing Nurses Knowledge of Glasgow Coma Scale in Emergency and Outpatient Department

**DOI:** 10.1155/2016/8056350

**Published:** 2016-12-01

**Authors:** Harvinderjit Kaur a/p Basauhra Singh, Mei Chan Chong, Hari Chandran a/l Thambinayagam, Mohd Idzwan bin Zakaria, Siew Ting Cheng, Li Yoong Tang, Nurul Hafizan Azahar

**Affiliations:** ^1^University Malaya Medical Centre, Kuala Lumpur, Malaysia; ^2^Department of Nursing Science, Faculty of Medicine, University of Malaya, Kuala Lumpur, Malaysia; ^3^Department of Surgery, Faculty of Medicine, University of Malaya, Kuala Lumpur, Malaysia; ^4^Faculty of Medicine Dean's Office, Faculty of Medicine, University of Malaya, Kuala Lumpur, Malaysia

## Abstract

Assessment of level of consciousness using the Glasgow Coma Scale (GCS) is a tool requiring knowledge that is important in detecting early deterioration in a patient's level of consciousness. Critical thinking used with the skill and knowledge in assessing the GCS is the foundation of all nursing practice. This study aims to explore the knowledge and competence in assessing the GCS among staff nurses working in the Emergency and Outpatient Departments. This is a quantitative descriptive cross-sectional study design using the GCS Knowledge Questionnaire. Convenience sampling method was used. Nurses in these Departments were asked to partake in the survey. Data collected was analyzed using the Statistical Package of Social Sciences (SPSS) version 20. Descriptive and Pearson's chi square was used. Result showed that 55.56% of nurses had poor knowledge followed by 41.48% and 2.96% with satisfactory knowledge and good knowledge, respectively. The result on the association between knowledge and education level showed a significant association between the two variables (*X* ^2^ = 18.412, df = 3, *n* = 135, and *p* < 0.05). There was also a significant correlation between knowledge and age group (*X* ^2^ = 11.085, df = 2, *n* = 135, and *p* < 0.05). Overall, this study supports that good knowledge and skill are important in assessing GCS level.

## 1. Introduction

Traumatic brain injury (TBI) is a leading cause of death and disability worldwide. Yearly, about 1.5 million people die from TBI and those several millions that survive receive emergency treatment [1]. In Malaysia, the statistics for the year 2009-2010 reveal that the causes of death from motor vehicle accidents are head injury (56.5%) followed by brain injury (38.1%), both head and brain injury (34%) and skull/craniofacial fractures (27.9%) [2]. The common presentation to the ED is with an acutely altered level of consciousness that requires quick assessment, which is the crucial action of all health providers [3, 4].

Consciousness has two components: arousal and content. Impairment of arousal can vary from mild (drowsiness or somnolence) to coma. Coma is the severest impairment of arousal and is defined as the inability to obey commands, speak, or open eyes to pain [5].

The important components to assess altered level of consciousness were designed in 1974 by Graham Teasdale and Bryan J. Jennettto, called the Glasgow Coma Scale (GCS) which tested 3 neurological aspects of the patient's response: eye opening, limb movement, and vocalization [5]. It is important to note that the scale is intended to assess level of consciousness and is not designed for following neurological deficits.

This tool is used worldwide for neurological assessment of level of consciousness in nursing practice and is further enhanced with the support of best practice guidelines. It is therefore the most sensitive and reliable indicator of all neurological patient's [6, 7]. Nurses who work in areas that care for these patients need to be competent in assessing GCS. The scoring will detect early deterioration in such patients [4]. Jaddoua et al. [8] showed that initial assessment of GCS obliviated unnecessary diagnostic tests and treatments.

## 2. Material and Methods

### 2.1. Study Design

This is a quantitative descriptive cross-sectional study design using convenience sampling. The sample size is calculated based on 95% confidence interval and with a margin error that does not exceed ±5 percent. 135 personnel participated in this study.

### 2.2. Setting

This study was conducted in the Emergency and Outpatient Departments of a Tertiary Medical Centre. The ED was divided into five areas, Red Zone (urgent cases) with 5 nurses per shift, Yellow Zone (acute cases) with 7 nurses per shift, Green Zone (semiacute cases) with 5 nurses per shift, Paediatric Emergency with 3 nurses per shift, and Trauma Ward with 4 nurses per shift. Outpatient Department which operates from 8 p.m. to 5 p.m. has 28 nurses on duty per day.

### 2.3. Selection of Participants

The eligibility criteria for this study were all nurses working, during data collection. Exclusion criteria include nurses on study leave, maternity leave, and sick leave.

### 2.4. Instrument

Questionnaires were used to collect data. It is divided into three parts. In Part A there are 4 questions related to demographic data addressing age, level of education, gender, and years of service. Part B consists of 15 multiple choice questions related to knowledge on Glasgow Coma Scale (GCS); see the following:

*Instrument to Assess the Knowledge on Glasgow Coma Scale*

(1) The Glasgow Coma Scale was initially devised to
  (a) locate brain tumour □
  (b) assess the depth of coma □
  (c) facilitate care for stroke patients □
  (d) monitor the extent of meningitis □
(2) What part of the brain is being assessed when you are assessing eye opening?
  (a) Cerebral cortex □
  (b) Occipital lobe □
  (c) Cerebellum □
  (d) Reticular formation □
  (e) Hypothalamus □
(3) Which part of the brain is being assessed when you are assessing verbal response?
  (a) Cerebral cortex □
  (b) Occipital lobe □
  (c) Cerebellum □
  (d) Reticular formation □
  (e) Temporal lobe □
(4) Which part of the brain is being assessed when you are assessing motor response?
  (a) Occipital lobe □
  (b) Cerebellum □
  (c) Sensorimotor pathways □
  (d) Dermatomes □
  (e) Reticular formation □
(5) What are the specific sections that comprise the Glasgow Coma Scale?
  (a) Eye opening, verbal response, pupil response □
  (b) Eye opening, verbal response, limb movement □
  (c) Eye opening, verbal response, motor response □
  (d) Eye opening, respiratory pattern, motor response □
  (e) Eye opening, respiratory pattern, pupil response □
(6) Vital signs are a component of the Glasgow Coma Scale.
  (a) True □
  (b) False □
(7) When testing the best motor response, you
  (a) Record the response in the best arm. □
  (b) Record the response in the worst arm. □
  (c) Record the best response from the legs. □
  (d) Record the response in all four limbs. □
(8) To test motor response in a tetraplegia patients (paralyzed in all four limbs),
  (a) Inflict a pain stimulus in the arms until there is a response. □
  (b) Inflict a pain stimulus in the legs until there is a response. □
  (c) Ask the patient to nod or turn his head. □
  (d) Lift the arm up and let it drop to the bed three times. □
(9) The lowest score of the Glasgow Coma Scale is
  (a) 1 □
  (b) 3 □
  (c) 4 □
  (d) 10 □
(10) Patients with a Glasgow Coma Scale score of—and below are considered comatose.
  (a) 1 □
  (b) 3 □
  (c) 8 □
  (d) 10 □
(11) In nursing practice, a reduction of the Glasgow Coma Scale score of—is seen asa deterioration in conscious level and requires informing the medical team.
  (a) 1 □
  (b) 3 □
  (c) 8 □
  (d) 10 □
(12) The Glasgow Coma Scale cannot assess intubated patient's level of consciousness.
  (a) True □
  (b) False □
(13) on asking a patient, “Do you know where you are now?” the patient states he is at his daughter's condominium. He is
  (a) Orientated. □
  (b) Confused. □
  (c) Producing inappropriate words. □
  (d) Producing incomprehensive sound. □
  (e) Is not responding. □
(14) On assessing a patient's motor response, he is unable to comply. You inflict a pain stimulus, and he pulls his arm away. He
  (a) Is obeying commands. □
  (b) Is localizing pain. □
  (c) Has abnormal flexion. □
  (d) Has abnormal extension. □
(15) You are assessing an RTA (road traffic accident) patient, who has swollen eyes. You instruct him to open his eyes, but he is unable to. The eye response score is
  (a) 4 □
  (b) C □
  (c) 2 □
  (d) 0 □

### 2.5. Validity and Reliability

The questionnaire used in this study is replicated from previous study in Singapore with permission. It has been tested for reliability and validity [9].

### 2.6. Data Collection and Analysis

Data was collected from July to September 2014. One hundred and thirty-five questionnaires were distributed, to nurses that met the inclusion criteria. Each nurse was given 15–20 minutes to answer the questionnaire, which was then returned. Data was analyzed using the Statistical Package of Social Sciences (SPSS) 20.0 version of window software. Descriptive and Pearson's chi square was used to test the assumption.

### 2.7. Ethical Considerations

Ethical approval was obtained from the Research Ethical Committee. Permission was given by nursing administration and nursing officials.

## 3. Results

135 questionnaires were distributed and all were returned culminating in a 100% respond rate.

### 3.1. Knowledge on GCS

88.9% of the nurses who participated knew what Glasgow Coma Scale was initially devised to. To the question pertaining to the part of the brain involved in assessing eye opening, 51.9% answer correctly as opposed to 31.1% for verbal response and 40.7% for motor response.

The majority (85.9%) answered correctly to the question, pertaining to the components of the Glasgow Coma Scale. Only 54.8% of the participants knew that vital signs are not a component of the Glasgow Coma Scale. Only about one-fifth (21.5%) of the nurses knew how to test the best motor response, but more than half (58.5%) knew how to test for motor response in a tetraplegic patient. While the vast majority (91.1%) knew what is the lowest score of the Glasgow Coma Scale, however only about half (51.9%) knew the score which defined comatose.

Only 11.9% responded correctly to the question on reduction of score to define deterioration. 63.7% said Glasgow Coma Scale can be assessed on an intubated patient's and 86.7% could answer the question pertaining to patients' verbal response. On assessing a patient's motor response with pain stimulus only 11.9% answered correctly but when assessing RTA (road traffic accident) patient, who has swollen eyes 84.4%, answered correctly.

This showed that the nurse's knowledge on GCS is poor in detecting deterioration of patient and in assessing the best motor response using pain stimulus.

### 3.2. Overall Knowledge Level of Nurses Working in Emergency and Outpatient Department

Overall only 2.96% had good knowledge, scoring is 80–100% (12–15 points), 41.48% had satisfactory knowledge, and the knowledge of 55.56% of the nurses who participated in the questionnaire was poor; that is, more than half had poor knowledge in assessing GCS.

### 3.3. Association between Knowledge and Demographic Variables

The result on the association between knowledge and education level shows that there was statistically significant (significant level is *p* value less than 0.05) association between the two variables (*X* ^2^ = 18.412, df = 3, *n* = 135, and *p* < 0.05) shown in Table 1. Therefore, this concludes that the two variables are associated. Nurses with certificates have good knowledge (25%) compared to post basic nursing (0%). This shows that skill and critical thinking are important in assessing GCS.

The result on association between knowledge and age group shows that there was a statistically significant (significance level is less than 0.05) association between the two variables (*X* ^2^ = 11.085, df = 2, *n* = 135, and *p* < 0.05) shown in Table 2. Therefore, this concludes that the two variables are associated. Nurses in age group of 41–60 had good knowledge (11.1%) compared to age group of 20–30 (1.1%). This shows experiences and skill is important when assessing GCS.

## 4. Discussion

Glasgow Coma Scale (GCS) is a reproducible tool used by nurses in almost every healthcare facility to assess level of consciousness in a patient with a neurological problem. It is important to have the skill and knowledge when assessing and applying critical thinking to interpret the findings.

Our survey showed 2.96% scored had knowledge, 41.48% had satisfactory knowledge, and 55.56% had poor knowledge of GCS. This is comparable to the finding by Teles et al. [7] who found that 74.55% of the staff nurses had average knowledge and 25.45% had poor knowledge in GCS, whereas Jaddoua et al. [8] showed that all nurses (*n* = 100) had inadequate knowledge in GCS.

Educational level is not the primary factor needed in assessing the GCS as shown in this study. The result on association between knowledge and education level shows that there was a statistically significant association between the two variables (*X* ^2^ = 18.412, df = 3, *n* = 135, and *p* < 0.05). Similar to the study by Heron et al. [10] on interrater reliability of the GCS found there were statistically significant differences with education qualification.

Skill comes in handy with experience as shown in this study. The result on association between knowledge and age group shows that there was statistically significant association between the two variables (*X* ^2^ = 11.085, df = 2, *n* = 135, and *p* < 0.05). Similar to the study by Heron et al. [10] on interrater reliability of the GCS found there were statistically significant differences with age.

The limitation of the study was that only nurses participated in this study. Further study should be conducted on all healthcare personnel practicing at the Emergency and Outpatient Departments.

## 5. Conclusion

This study found that only 2.96% of nurses have good knowledge in GCS. This finding raises concerns on the importance of knowledge and skill in assessing GCS. Continuing education and practice on use of the GCS tool are important.

Education and age have a correlation with satisfaction level towards nurse's knowledge in GCS. This indicates that midage nurses with lower education level have higher skill and experience on using the GCS tool.

## Figures and Tables

**Table 1:** Pearson's chi square association between knowledge and level of education (*n* = 135).

Level of education	Knowledge				Pearson's chi square		
	Good	Satisfactory	Poor	Total	*X* ^2^	df	Sig. (*p*)
Diploma	1 (1.1%)	34 (36.2%)	59 (62.8%)	94 (100%)	18.412^a^	3	0.005
Post basic nursing	0 (0%)	11 (57.9%)	8 (42.1%)	19 (100%)			
Degree	2 (11.1%)	10 (55.6%)	6 (33.3%)	18 (100%)			
Certificate	1 (25%)	1 (25%)	2 (50%)	5 (100%)			
Total	4 (3%)	56 (41.4%)	75 (55.6%)	135 (100%)			

^a^6 cells (50%) have expected count less than 5. The minimum expected count is 0.

**Table 2:** Pearson's chi square association between knowledge and age group (*n* = 135).

Age group	Knowledge				Pearson chi square		
	Good	Satisfactory	Poor	Total	*X* ^2^	df	Sig. (*p*)
20–30	1 (1.1%)	38 (42.2%)	51 (56.7%)	90 (100%)	11.085^a^	2	0.026
31–40	1 (3.7%)	15 (55.6%)	11 (40.7%)	27 (100%)			
41–60	2 (11.1%)	3 (16.7%)	13 (72.2%)	18 (100%)			
Total	4 (3%)	56 (41.4%)	75 (55.6%)	135 (100%)			

^a^4 cells (33.3%) have expected count less than 5. The minimum expected count is 1.

## References

[1] Bruns J., Hauser W. A. (2003). The epidemiology of traumatic brain injury: a review. *Epilepsia*.

[2] Sabariah Faizah J., Mahathar A. W., Mohd Yusof A. W., Yeoh T. M., Ismail M. S. National Trauma Database January 2009 to December 2009-Fourth Report, Malaysia 2011. http://www.acrm.org.my/ntrd.

[3] Kevric J., Jelinek G. A., Knott J., Weiland T. J. (2011). Validation of the Full Outline of Unresponsiveness (FOUR) scale for conscious state in the emergency department: comparison against the Glasgow coma scale. *Emergency Medicine Journal*.

[4] Nguyen T. H., Sun-Mi C. (2011). The accuracy of Glasgow coma scale knowledge and performance among Vietnamese nurses. *Perspectives in Nursing Science*.

[5] Matis G., Birbilis T. (2008). The Glasgow Coma Scale—a brief review. Past, present, future. *Acta Neurologica Belgica*.

[6] Gocan S., Fisher A. (2008). Neurological assessment by nurses using the National Institutes of Health Stroke Scale: implementation of best practice guidelines. *Canadian Journal of Neuroscience Nursing*.

[7] Teles M., Bhupali P., Madhale M. (2013). Effectiveness of self instructional module on knowledge and skills regarding use of Glasgow coma scale in neurological assessment of patients among nurses working in critical care units of KLE Dr. Prabhakar Kore hospital and medical research centre, Belgaum. *Journal of Krishna Institute of Medical Sciences University*.

[8] Jaddoua B. A., Mohammed W. K., Abbas A. D. (2013). Asesment of Nurse's knowledge concerning glasgow coma scale in neuro surgical wards. *Journal of Kufa for Nursing Scienc*.

[9] Mattar I., Liaw S. Y., Chan M. F. (2013). A study to explore nurses' knowledge in using the Glasgow coma scale in an acute care hospital. *Journal of Neuroscience Nursing*.

[10] Heron R., Davie A., Gillies R., Courtney M. (2001). Interrater reliability of the Glasgow coma scale scoring among nurses in sub-specialties of critical care. *Australian Critical Care*.

